# A Study on the Cause Analysis of Cyberbullying in Korean Adolescents

**DOI:** 10.3390/ijerph17134648

**Published:** 2020-06-28

**Authors:** Woochun Jun

**Affiliations:** Department of Computer Education, Seoul National University of Education, Seoul 06639, Korea; wocjun@snue.ac.kr; Tel.: +82-2-3475-2504

**Keywords:** cyberbullying, adolescent, cybercrime, verbal abuse, instant message, information and communication technology, information ethics

## Abstract

With the development of information and communication technology, online communication is becoming more active than offline meetings in daily life. This online communication is accelerating, especially as smartphone distribution and utilization become more prevalent. This communication in cyberspace has the advantage of people being able to communicate anytime, anywhere beyond time and place, while causing a variety of inappropriate consequences. A typical one is cyberbullying, which is a serious problem for adolescents who have active communication online. The purpose of this study is to accurately investigate and analyze the status of cyberbullying among adolescents. To this end, national survey data of the National Information Society Agency (NIA) was analyzed for the past three years. The population size and sample size from 2017 to 2019 were 5.773.998 and 4500 (2017), 5,663,725 and 4662 (2018), 5,502,801 and 4779 (2019), respectively. The statistical analysis shows that the biggest type of cyberbullying among adolescents is verbal abuse, and the biggest means is instant messaging. In addition, the most frequent forms of cyberbullying victims and cyberbullying perpetrators occur between individuals. In addition, the correlation between the interpersonal relationships of adolescents and the cyberbullying experience rate were analyzed, and various cyberbullying factors such as psychological factors were analyzed. As a result, we found that the interaction with parents and friendship reliability have a negative correlation with the cyberbullying experience rate. We expect the results of this study to be of great help to future research and policies of juvenile cyberbullying.

## 1. Introduction

Modern society is a knowledge information society, and the core technologies of a knowledge information society are smart technology, information technology, and communication technology. Smart technology aims to simulate a system by giving sensing, control, and computation functions to mechanical, aeronautical, and civil structures so that they can detect changes in their surroundings and respond to information in real-time [1]. On the other hand, information and communication technology (ICT) is “an extensional term for information technology (IT) that stresses the role of unified communications and the integration of telecommunications and computers, as well as necessary enterprise software, middleware, storage, and audiovisual systems that enable users to access, store, transmit, and manipulate information” [2].

Smart technology, along with information and communication technology, provided abundant benefits to our lives in a modern knowledge and information society. Among the various benefits of information and communication technology, online communication is especially a very convenient benefit. In other words, offline meetings are limited in time and place, but online meetings in virtual space can transcend time and place, giving and receiving real-time feedback anytime, anywhere. This online communication not only allows individuals to communicate actively, but also causes socially active communication, which has the advantage of being able to inform adolescents quickly in case of an emergency, such as an earthquake, and also to quickly gather various opinions on school life issues.

However, while information and communication technology provides us with various benefits, it also causes various negative impacts. Among the various negative impacts, cyberbullying is a typical one, causing us not only mental but also social and economic problems. Especially for adolescents, whose values and personalities have not been established yet, deviations from cyberspace cause a number of problems, including emotional problems and academic interference. With the popularization of smartphones, adolescents increasingly own and use smartphones. Thus, cyberbullying can be a more serious social phenomenon in the future for adolescents, and it requires proper prevention and treatment.

The purpose of this study is to investigate and analyze the cyberbullying status of adolescents. Specifically, the purpose of this study is to analyze the occurrence process, along with the causes of cyberbullying among adolescents. In order to do this, it is necessary to investigate and analyze the cyberbullying status of various adolescents correctly, accurately, and objectively. These vast surveys require a lot of time and effort, and there should be proper questionnaires that are done as fairly as possible.

In this study, we decided to use the nation-level cyberbullying survey reports to analyze the cyberbullying status of adolescents as fairly and objectively as possible. Since 2014, the National Information Society Agency (NIA, http://www.nia.or.kr) has conducted a comprehensive and systematic survey of cyberbullying and analyzed the results statistically [3,4,5]. In addition, NIA has conducted and published a survey on the public’s internet use [6,7,8,9,10,11,12,13,14,15,16,17]. The study analyzed the cyberbullying status of adolescents based on cyberbullying reports for three years from 2017 to 2019. The population size and sample size from 2017 to 2019 were 5.773.998 and 4500 (2017), 5,663,725 and 4662 (2018), 5,502,801 and 4779 (2019), respectively. The purpose of using the statistics for the last three years is to include the most recent statistics, which are different from previous years’ questionnaires. In this work, adolescents include elementary school students, middle school students, and high school students, respectively.

In this study, based on the survey results, we show whether the following three hypotheses are correct or not.

**Hypothesis 1** **(H1).** *The most common type of cyberbullying is verbal violence.*

**Hypothesis 2** **(H2).** *The most common means of cyberbullying is instant messaging.*

**Hypothesis 3** **(H3).** *The most common form of cyberbullying occurs between individuals.*

## 2. Related Works

### 2.1. Introduction of Cyberbullying

In the literature, definitions and characteristics of cyberbullying are as follows.

According to [18], cyberbullying is defined as “an act or a phenomenon that persistently harasses a specific person in the cyber world”. In other words, it refers to the act of constantly harassing an opponent by using smartphone messengers, mobile phone text messages, and other online communication tools such as SNS (Social Network Service), emails, and electronic bulletin boards. On the other hand, when an act of cyberbullying is further expanded, it can spread false information about the victim or expose the victim’s personal information to illegal or obscene sites such as sex trafficking sites. Such online verbal abuse and slander are difficult to delete completely because so many people watch and spread the information at the same time. It is also a serious social problem that is spread widely in a short period of time, causing visual shock from videos and composite pictures.

The “Act on Prevention and Countermeasures of School Violence”, as amended on 19 November 2014, defines cyberbullying as “any act that causes students to suffer by continuously and repeatedly using information and communication devices such as the internet, mobile phones, or spreading personal information or false information related to specific students” [19].

In [3], eight types of cyberbullying are described as follows.

1. Cyber verbal violence

The act of swearing, harsh language, and personal offensive remarks through the internet or mobile phone texting services.

2. Cyber defamation

The act of posting defamed articles on the internet or social media, regardless of whether they are true or not, so that anyone (an unspecified majority) can see them.

3. Cyber stalking

The act of repeatedly sending emails or text messages (letters) that cause fear or anxiety, or visiting blogs or SNSs (Social Network Services) to leave a trace of comments, even though certain people do not want to do so.

4. Cyber sexual violence

The act of posting sexually offensive content, such as sexual depiction, sexually disparaging remarks, and sexist swearing, on the internet or on mobile phones or spreading obscene videos or photos of certain people.

5. Personal information leakage

The act of referring to or posting personal and private details or secrets on the internet or social media or disseminating personal information (e.g., name, residence, school)

6. Ostracism

The act of ostracizing someone in an internet chat room, on smartphones, or through instant messaging.

7. Cyber extortion

Stealing cyber money or smartphone data from the internet.

8. Cyber coercion

The act of forcing someone on the internet to run errands for someone else or to speak or act in a way that is unwanted by the person.

Cyberbullying includes malicious comments or messages, demeaning or humiliating photos or composite photos, and all acts that can cause shame or distress to the other party, such as posting video clips or text (message) bombs (that is an act of sending many text messages at once), mass blocking of messengers, collective rejection of friend requests, stalking, and spreading false information. This cyberbullying has a variety of characteristics because it is done in a virtual space called cyberspace, unlike the real world where bullying is directly face-to-face.

As cyberbullying is done through the various services provided by digital devices, it is done in a wide variety of formats and methods, depending on the different types of communication that digital services provide. In addition, the spread is very fast as it is not only immediately delivered to the victim according to the network attributes that connect in real-time, but also, once posted, verbal abuse or slander spreads quickly through cloning without time or place constraints.

Cyberbullying can be done anytime via email, mobile phone, or SNS (Social Network Service), so victims are likely to suffer indiscriminate violence around the clock. Added to this are audiovisual shocks such as videos, photos, and voice recordings; the intensity of the violence becomes even greater. With cyberbullying, it is also difficult to determine who the perpetrator actually is because the perpetrator can steal an identity from an internet service or open an anonymous email address to hide his or her identity.

It is also more likely that cyberbullying will develop a more brutal aspect as it is difficult for the perpetrators to accurately recognize how much pain the victim suffers because they are not face-to-face. In particular, cyberbullying among adolescents is difficult for adults, such as parents and teachers, to detect properly, so it is difficult to recognize that the victim is suffering unless he or she directly reveals the damage.

According to [20], cyberbullying has the following characteristics. First, it has anonymity and a non-face-to-face character. Cyberbullying assailant identities are anonymous, and the features of cyberbullying prevent them from seeing emotional reactions from the victims, such as anger, anxiety, and nervousness. For this reason, even inadvertent, accidental cyberbullying cannot be immediately contained. Conversely, face-to-face communication, where one can see the changes in the victims’ emotions, can be adjusted according to the other person’s reaction. When teased offline, even the perpetrator’s nonverbal communication provides a social clue, so its intent can be read. In cyberspace, lack of face-to-face communication leads to fewer opportunities for social clues. Anonymity and the non-face-to-face feature can make it easier to forget the human aspects of the cyberbully’s target, making the perpetrator more aggressive. Second, the perpetrator has accessibility to the victim beyond time and place. Cyberspace can be accessed anytime and anywhere by an assailant as long as the victim’s electronic devices are powered on. Cyberbullying and offline bullying both have a negative impact on the victim, but victims of traditional bullying could at least take a breather if they leave the bullying scene. However, cyberbullying victims feel that there is no safe place 365 days a year. Victims can turn off their computers or cell phones, but as soon as they turn their devices back on, nightmarish writing and text appear again. Cyberbullying is likely to continue as “no-stop bullying” because neither the perpetrator nor the victim is bound in physical space and time. The third characteristic is an extension of real violence. Cyberbullying occurs mostly in conjunction with reality because cyberspace and real space are on a continuous line. The non-face-to-face cyberspace induces acts of revenge for other person’s provocation more easily than in reality. Sometimes, revenge for a real experience is realized in cyberspace. The final characteristic is the difficulty in recognizing the damage. In a traditional bullying situation among adolescents, the perpetrator threatens the victim not to inform his parents or teacher. With cyberbullying, however, is easy to hide the fact that the victim is being bullied. This is because the victim himself needs to be isolated from the digital device first when help is notified. Thus, cyberbullying often leads to very serious situations that parents or teachers are unaware of.

### 2.2. Previous Works

This section introduces previous research on causes related to cyberbullying. There has been some research work for causes of cyberbullying in adolescents, as follows.

#### 2.2.1. Cases in Korea

In [21], the effects of elementary school students’ cyber victimization on cyberbullying behavior, and the mediating and moderating effects of anxiety- and stress-coping strategies were investigated. Data were collected for 970 students in Grades 4–6 in elementary school, and structural equation model analysis and hierarchical regression analysis were performed. The main research results are as follows. First, it was shown that elementary school students’ cyber victimization experience can increase cyberbullying behavior. Second, the mediating effect of anxiety and the moderating effect of rational coping strategies were verified in the relationship between cyberbullying victimization and cyberbullying behavior. The results of this study showed that in order for cyber victimization not to lead to cyberbullying, counseling and training are needed to ease anxiety.

Kwon and Kim aimed to look at the variables affecting cyberbullying of adolescents, which should be given more attention due to social problems, in terms of personal, domestic, and school environments, and digital media factors, and more specifically, to analyze the differences in the cause variables by dividing the students by gender [22]. The subjects of this study were middle and high school students with smartphones, who used online (email and smartphone) response methods using random allocation extraction methods nationwide. The survey was conducted for eight days in August 2018, and a total of 811 responses were adopted, after eliminating unfaithful responses. The characteristics of the 811 teenagers are as follows: By gender, the numbers were similar, with 391 male students (48.2 percent) and 420 female students (51.8 percent). By school, 322 middle school students (39.7%) and 489 high school students (60.3%) were included. Parents had the largest number of high school graduates as academic background, with 64.7 percent of dual-income households. The results presented through technical statistics, T-test, correlation analysis, and multiple regression analysis can be summarized as follows. First, the technical statistics showed that male student cyberbullying scores were higher than female student scores, and the T-analysis results were also statistically significant. Second, multiple regression analysis, including gender variables, showed that cyberbullying by male students was higher than that of female students. Finally, the multiple regression analysis of male and female students was conducted, and the variables affecting cyberbullying behaviors of male and female students were somewhat different. Common cause variables were parental stress, peer affection, and game addiction, which were statistically significant static (+) directions. Different factors of the cause were depression, aggression, and SNS addiction among boys, while female students were under academic stress, suggesting differences in cyberbullying factors depending on gender.

The work by Woo, Kwak, and Lee was conducted to identify the medium effects of aggression in the relationship between high school students’ overuse of smartphones and cyberbullying. In total, 328 high school student responses were collected and analyzed using self-reporting questionnaires [23]. In this study, the analysis found that cyberbullying has a static correlation with the overuse of smartphones and aggression. Overuse of smartphones has a direct impact on aggression, and aggression has a direct impact on cyberbullying. In addition, the overuse of smartphones has had an indirect effect on cyberbullying by using aggressiveness as a medium. In order to reduce cyberbullying among high school students, it is thought that it will be necessary to develop cyberethics education programs to reduce aggression, along with regulations on the overuse of smartphones.

Jung and Park verified the parametric effects of self-control on the impact of the cognitive and emotional empathy of adolescents on cyberbullying [24]. To this end, 254 middle school students were measured for empathy, self-control, and cyberbullying experiences. The results are as follows. First, the higher the cognitive empathy, the higher the level of self-control, and the less cyberbullying. In addition, the higher the level of self-control, the less cyberbullying. Second, emotional empathy did not show a significant correlation with both cyberbullying and self-control. Third, it was shown that the cognitive empathy of adolescents does not directly affect cyberbullying, but indirectly by means of self-control.

In [25], the purpose of the study was to find out the psychological characteristics of teenagers and the impact of cyber victimization experiences on cyberbullying behavior. For this research, 625 teenagers attending middle and high schools were asked about cyberbullying behavior, cyber victimization experience, depression, anxiety, and anger. The main results of this study are as follows. First, significant variables that predict cyberbullying behavior among teenagers were found to be in the order of cyberbullying experience, gender, and anger. Second, the adolescents’ experience of cyberbullying showed a fully mediated effect on the effects of depression or anxiety on cyberbullying behavior, while the effects of anger on cyberbullying behavior were partially mediated.

Shin analyzed the impact of teenagers watching violent actions and violence against the school and cyberbullying and verified the effect of self-control on these impacts [26]. The results of the study are the following: First, the more violent targets teenagers have, the more likely they are to commit school violence and cyberbullying. Second, looking at the direct effects of self-control used as parameters, the lower the self-control, the higher the likelihood of school violence and cyberbullying. Third, both the target of violence and the viewing of violent actions have significant effects on school violence and cyberbullying through the use of self-control.

In [27], the study was aimed at investigating the prevalence of cyberbullying and the perpetuation factors of cyberbullying through a national sample of 4000 youths selected through multistage cluster sampling. The respondents were 2166 male students and 1834 female students in the 7th and 12th grades of 24 middle and high schools nationwide. The statistical analysis of the survey data is summarized as follows: First of all, 34 percent of the respondents were involved in cyberbullying as cyberbullies (6.3 percent), victims (14.6 percent), and both cyberbullies and victims (13.1 percent). Boys were more likely to be cyberbullies than girls. Second, temporal variables due to chat services, social networking services (SNS), cyberbullying experiences, and offline bullying practices tended to increase the probability of students becoming perpetrators of cyberbullying. However, cognitive empathy variables contributed to reducing the permanent behavior of cyberbullying. Third, the variables of parental attachment and satisfaction with school life had little effect on the perpetuation of cyberbullying.

#### 2.2.2. Cases of Other Countries

Kim, Colwell, Kata, Boyle, and Georgiades investigated the association between cyberbullying and adolescent mental health problems [28]. The study also examined the extent to which this association differs by gender and mental health problem type. For a survey study, a total of 31,148 students in grades 6–12 participated in an anonymous type of survey. They were asked for their frequency of exposure to traditional forms of bullying, cyberbullying, and experiences of mental health problems over the past 6 months, and multilevel structural equation modeling was adopted to examine the associations. The following results are obtained. Cyberbullying was more strongly associated with emotional problems for girls and with behavioral problems for boys. The result means that cyberbullying causes both emotional and behavioral problems for adolescents.

In [29], the study was aimed at exploring the relationship between traditional and cyberbullying damage, along with self-reported health and life satisfaction, and to investigate whether engaging in risky behavior contributes to these health outcomes. A total of 318 students aged 15 to 18, attending eight elementary schools in Ireland, completed the survey. They were asked to answer questions about traditional bullying and cyberbullying, risky behavior, and self-report health and life satisfaction as school children. Children who were victims of bullying were more likely to report being in poor health and less satisfied with life and having more dangerous behavior. While not statistically significant, it is found that cyberbullying has a positive bearing on increased reporting of poor health and low life satisfaction. Traditional bullying is the most common form of bullying among school children in Ireland and seems to have a stronger connection with poor overall health. However, many children are victims of cyberbullying and traditional violence. They concluded that it is important to acknowledge, identify, and deal with all kinds of bullying to improve children’s health outcomes.

Based on a 2013 survey of 9512 ninth-grade students in Lower Saxony, Germany, a paper examined the correlation between the prevalence of cyberbullying and these behaviors [30]. Binary logistic multistage regression analysis was used to analyze the correlation between sexual and psychological cyberbullying. In the preceding semester, 2.4 percent of adolescents were psychological, cyberbullying perpetrators, and 0.4 percent had sexually harassed someone online. Low levels of empathy, frequent violent media consumption, and victimization of aggressive online behavior are linked to the risk of child bullies. Female teenagers are less likely to engage in sexual cyberbullying than boys, but more likely to engage in psychological cyberbullying. Only a small number of teenagers were involved in sexual and psychological cyberbullying. The correlation between the two behaviors is different, but being a victim of aggressive online behavior increases the victimization of both behaviors.

Udris analyzes cases of cyberbullying among adolescents in Japan. Participants included 899 high school students who wrote their own report questionnaire on technology usage habits, cyberbullying, and cyber victim experiences [31]. Using logistic regression analysis, the relationships between several independent variables were measured, including cyberbullying and cyber victimization and gender, age, and technology use. The survey found that 22 percent of the participants had experienced cyber victimization. Also, 7.8 percent of people admitted that they bullied others. Most cases of cyberbullying knew the identity of their bully as classmates and victims. Multiple logistic regression found that cyber victimization is the biggest predictor of cyber-bullying and vice versa. Having more online friends has a lot to do with cyberbullying and cyber victimization.

Hellfeldt, Lopez-Romero, and Andershed experimented with the relationship between cyber-bullying roles and several psychological well-being outcomes and the potential mediation effects that social support perceived by family members, friends, and teachers in schools could bring about [32]. This was investigated in a cross-sectional sample of 1707 youths (girls 47.5 percent, aged 10 to 13 years old, self-reporting through web questionnaires) who were attending community and private schools in a Swedish mid-sized local government district. They concluded from the results that cyberbullying victim groups have the highest levels of depression and the lowest level of subjective well-being and family support. They also observed higher levels of anxiety symptoms in both cyber-victims and cyberbully-victims. Moreover, they concluded that some form of social support appears to be protective in the way it mediates the relationship between cyberbullying and psychological well-being. Specifically, social support perceived by families and teachers lowers the probability of depression and anxiety, and the higher the level of family social support, the higher the level of subjective happiness for teenagers that are cyber-victims and cyberbully-victims.

Romero-Abrio, Martinez-Ferrer, Musitu-Ferrer, Leon-Moreno, Villarreal-Gonzalez, and Callejas-Jeronimo analyze the relationship between family communication problems and cyberbullying through mental and social adjustments, such as psychological distress, attitude towards institutional authorities, and problematic use of social networking sites for adolescents [33]. In this study, random sampling by conglomerates was conducted. A total of 8115 teenagers participated in the study (51.5% for boys and 49.5% for girls), aged 11 to 16 (M = 13.34, SD = 1.04) and registered in Nuevo Leon (Mexico). The structural equation model was developed using structural equation modeling software (EQS). The results showed that problematic family communication was directly linked to cyberbullying, and also indirectly related to the use of social networking sites through the relationship of psychological distress and attitudes toward violations of social norms. A multigroup analysis also revealed gender differences in these relationships.

## 3. Statistical Analysis of Cyberbullying in Adolescents

In this section, we present various statistical analyses on cyberbullying of adolescents in Korea as follows.

### 3.1. Analysis Methods

The purpose of the statistical analysis is to identify the various causes of cyberbullying in adolescents. In this study, we would like to find out the following specific conditions regarding the cyberbullying causes of adolescents. First, it is about the type of cyberbullying. Second, it is about the means of cyberbullying. In other words, we look at the means of communication through which cyberbullying occurs. Third, it is to find out whether cyberbullying occurs between groups or between individuals.

For the statistical analysis of this work, we adopted nationwide statistical data from the National Information Society Agency (http://www.nia.or.kr) [3,4,5]. The agency has announced cyberbullying statistics since 2014. Samples of this survey have been collected evenly throughout the country. The survey results since 2017 were analyzed using the Statistical Package for the Social Sciences (SPSS) WIN 25.0 program (IBM, Armonk, NY, USA). One-way ANOVAs and *t*-tests were conducted to find out the type, means and forms of cyberbullying by adolescents. Note that the survey method before 2017 was different from the survey method after 2017, so data before 2017 was not adopted.

The purpose of the national cyberbullying survey by NIA is to identify the status of cyber violence and the level of instructional education through quantitative investigation and to secure in-depth evidence, such as cases by type of cyber violence, through qualitative investigation. The survey was conducted as follows. In this paper, we introduce the most recent data of 2019 since the survey was carried out for the rest of the year in a similar way. First, the survey period was about six weeks, from 1 October to 23 November 2019. In addition, 4779 samples were selected from a population of about 5.5 million, and data were collected through mail, internet surveys (quantitative surveys), and collective interviews (qualitative surveys). Meanwhile, sampling methods were used for a stratified extraction method (quantitative survey) and random sampling (qualitative survey). The sample error (95% confidence level) was ±1.42% *p*. The survey items included internet usage behavior, cyber victimization experience, cyberbullying experience, cyberbullying witness experience, and social and psychological environment factors.

### 3.2. Cyberbullying Survey Data

The cyberbullying status of adolescents based on the National Information Society Agency [3,4,5] is summarized as follows.

First, the following Table 1 and Table 2 show the cyberbullying status of adolescents by type.

The following Table 3 and Table 4 show the cyberbullying status of adolescents by means.

The following Table 5 and Table 6 show the cyberbullying status of adolescents by forms.

As we can see from Table 1 and Table 2, in the cyberbullying of adolescents, the most common type of violence is verbal violence. Moreover, from Table 3 and Table 4, we can see that the most common means of violence is instant messaging. From Table 5 and Table 6, we can see that the most common form of violence occurs between individuals.

### 3.3. Statistical Analysis Results

The results of the statistical analysis for cyberbullying type are shown in Table 7.

As shown in Table 7, the average value of each type of cyberbullying is as follows. Verbal violence is the highest at 16.90, followed by defamation 4.80, stalking 2.33, personal information leakage 2.25, ostracism 1.80, sexual violence 1.72, extortion 1.20, and coercion 0.87. The statistically significant difference is F = 188.38, *p* < 0.001. Therefore, we can conclude that the biggest type of cyberbullying among adolescents is verbal violence.

In addition, the results of statistical analysis for cyberbullying means are shown in Table 8.

As shown in Table 8, the average value for cyberbullying means is as follows. Instant messages are the highest at 48.62, followed by online games 39.50, SNS 30.47, community 4.00, email/text 2.52, and personal homepage 2.08. The statistically significant difference is F = 259.14; *p* < 0.001. Therefore, the biggest means of cyberbullying among adolescents is instant messaging.

Finally, the results of statistical analysis for cyberbullying forms are shown in Table 9.

As shown in Table 9, the mean for person-to-person violence is 73.88, higher than the 25.62 mean for group-to-person violence (t = 28.26, *p* < 0.001). Thus, it is shown that cyberbullying among adolescents occurs more in a person-to-person fashion than in a group-to-person manner.

### 3.4. Nonparametric Statistical Analysis Results

This section introduces the results of applying nonparametric statistical methods to analyze survey data in various ways. In this study, Kruskal–Wallis H verification and Mann–Whitney U verification were performed among nonparametric statistical techniques.

The results of statistical analysis for cyberbullying type are shown in Table 10.

As shown in Table 10, verbal violence is the highest among the types of cyberbullying with 45.50, followed by defamation with 39.17, personal information leakage with 28.00, stalking with 26.00, ostracism with 20.17, sexual violence with 18.50, extortion with 10.50, and coercion with 8.17. There is a significant difference in statistics (Χ^2^ = 36.48, *p* < 0.001). Therefore, it can be seen that the most common type of cyberbullying among adolescents is verbal violence.

Additionally, the results of statistical analysis for cyberbullying means are shown in Table 11.

As shown in Table 11, instant messaging tops the list of cyber-bullying means with 33.50, followed by online gaming 27.50, SNS 21.50, community 13.00, email/text 8.42, and personal homepages 7.08, showing a statistically significant difference (Χ^2^ = 31.24, *p* < 0.001). Therefore, it can be seen that the most common means of cyberbullying among adolescents is instant messaging.

Finally, the results of statistical analysis for cyberbullying forms are shown in Table 12.

As shown in Table 12, the average ranking is 9.50 for person-to-person violence, which is higher than 3.50 for group-to-person violence, with a statistically significant difference (Z = -2.88 and *p* < 0.01). Therefore, it can be seen that cyberbullying among adolescents occurs more in a person-to-person way than in a group-to-person way.

### 3.5. Correlation Analysis of Parent and Friend Relationships

In this section, to analyze the various causes of adolescent cyberbullying, the factors of parent and friend relationships are analyzed. To this end, the latest adolescent cyberbullying status report [3] is used to analyze three factors as follows.

1. The relationship between parents’ involvement in internet use and their children’s cyberbullying experience rate.
2. The relationship between parents’ and children’s interaction with their children’s cyberbullying experience rate.
3. The relationship between friend relationship reliability and the adolescents’ cyberbullying experience rate.

The collected data in [3] were analyzed using the SPSS WIN 25.0 program. Correlation analysis was conducted to find out the relationship between the adolescent cyberbullying experience rate and the three factors.

Table 13, Table 14 and Table 15 show the parents’ involvement in internet use and their children’s cyberbullying experience rate, the interaction between parents and children and their children’s cyberbullying experience rate, and the reliability of friend relationships and the cyberbullying experience rate of adolescents, respectively.

To analyze the correlation, the following three hypotheses are established:

**Hypothesis 4** **(H4).** *Parents’ internet involvement and cyberbullying experience rates are negatively correlated.*

**Hypothesis 5** **(H5).** *Interaction between parents and children and their cyberbullying experience rates are negatively correlated.*

**Hypothesis 6** **(H6).** *Reliability of friendship relationships and cyberbullying experience rates are negatively correlated.*

The results of the correlation analysis to analyze whether the above three hypotheses are established are shown in Table 16, Table 17 and Table 18, respectively. In Table 16, Table 17 and Table 18, the experience rate includes both the rate of cyberbullying and the rate of cyber victimization.

Parents’ involvement in internet use shows no statistically significant correlation with the adolescents’ cyberbullying experience (r = −0.896, *p* > 0.05). Therefore, it can be seen that Hypothesis 4 cannot be adopted.

Interaction between parents and children shows a statistically significant negative correlation with cyberbullying experience rates (r = −0.970, *p* < 0.05). That is, the higher the interaction with parents, the lower the cyberbullying experience rate. Therefore, it can be seen that Hypothesis 5 is supported.

Reliability of the friend relationship shows a statistically significant negative correlation with the cyberbullying experience rate (r = −0.994, *p* < 0.01). In other words, the higher the reliability of the friend relationship, the lower the cyberbullying experience rate. Therefore, it can be seen that Hypothesis 6 is supported.

### 3.6. Analysis of Various Factors of Cyberbullying

In this section, we discuss the reasons for cyberbullying in adolescents in various ways.

According to [3], the reasons for cyberbullying are those shown in Table 19. As shown in Table 19, 45.0% of the respondents said, ‘The other party did it first, to retaliate’ as the reason for cyberbullying behavior in 2019. In addition, the number of adolescents who say that the other party did it first (to retaliate) has increased every year since 2017.

In addition, Table 20 shows the post-abuse psychological state, according to [3]. It can be seen that 51.9 percent of students who committed cyberbullying felt “sorry and regretful,” while 49.0 percent were “worried about having problems”.

As Table 19 and Table 20 show, the cyberbullying is impulsive and an immediate retaliation when he or she is victimized. It can also be concluded that once an attack occurs, the cyberbullying perpetrator regrets his or her actions or fears the occurrence of subsequent events.

Meanwhile, let us take a look at the victim’s response and psychological status. First, Table 21 shows the victim’s response, where 36.6 percent of students who experienced cyberbullying experienced “blocking the other person or deleting or changing their own IDs or emails”, while 26.7 percent responded that “the other person was asked to delete the abuse or to apologize directly”.

Meanwhile, Table 22 shows why the victims did not respond after the cyber victimization. Of the reasons for not doing anything after experiencing cyber victimization, in 2019, 75.0 percent said they thought it was nothing, up from 9.1 percent in 2018.

Table 23 shows the psychological states after the victimizations. In 2019, after experiencing cyber victimization, 54.0 percent of students said they did not think much about it. In addition, 36.3 percent of them felt “the desire for revenge on the other party”, and 20.9 percent experienced “depression, anxiety, and stress”.

Furthermore, the following Table 24 shows cyberbullying and cyber victimization experience rates by school and gender [3]. Note that both rates mean both cyberbullying and cyber victimization experience rates.

As Table 24 shows, cyberbullying and victimization experiences among elementary, middle, and high school students decreases in the order of middle school, high school, and elementary school students. Additionally, by gender, male students have a higher experience rate of both cyberbullying and cyber victimization than female students.

## 4. Discussion

A modern knowledge and information society provides us with many benefits. Among the various benefits we enjoy, online meetings in cyberspace using information and communication technology and smart communication technology are particularly useful for us. In other words, we can exchange or share various opinions through these communication tools. However, this online communication creates not only advantages but also various negative impacts. The most common of these negative impacts is cyberbullying, especially for adolescents whose information and communication ethics are not properly established.

As a result of the analysis of cyberbullying so far, the following results have been obtained. First, the most common type of cyberbullying in adolescents is verbal violence. Second, the most common means of cyberbullying is instant messaging. Third, the most common form of cyberbullying occurs between individuals. Fourth, the interaction between parents and children show a statistically significant negative correlation with cyberbullying experience rates. Fifth, the reliability of friend relationships shows a statistically significant negative correlation with cyberbullying experience rates. Additionally, from Table 21, Table 22 and Table 23, we can see that victims are shown to be passive in their response to victimization. Moreover, the victims were either afraid or too passive to report their own abuse. The victims, meanwhile, can be seen as trying to ignore the fact of the damage to themselves.

The above results imply the following. First, cyberbullying occurs spontaneously among adolescents. The common characteristics of cyberbullying are summarized as verbal violence, instant messaging, and communication between individuals. In other words, their common characteristic is that communication among adolescents can be improvised in real-time. Adolescents can be considerate in an environment where they can afford to think, even a little, but in real-time media space, they have no choice but to improvise. Second, adolescents who are victims of cyberbullying are overly passive about cyberbullying and even ashamed of their own abuse. This can be attributed to the social perception that it is the victim’s fault for providing the cause of cyberbullying to some extent. Third, cyberbullying perpetrators are insensitive to their own cyberbullying behaviors and do not regret their actions. This can be attributed to the fact that there is anonymity, due to the nature of cyberspace, and that they can hide themselves, making them bolder than in the real world. Fourth, improving the interpersonal skills of adolescents, such as improved interaction with parents and improved reliability of friend relationships, can reduce cyberbullying and cyber victimization experiences.

Therefore, in order to solve cyberbullying in adolescents, we propose the following. First, the best way to solve cyberbullying in adolescents is prevention, not healing. What is needed most of all for prevention is to strengthen information and communication ethics education for them. It is necessary to provide after-school programs and various special lectures, as well as public education provided by schools. Second, various means for cyberbullying victims to report their damages justly should be provided. In other words, they should be able to report the abuse quickly through online civil petitions, and they should also know the process of handling their report transparently. Third, adolescents should be encouraged to use non-real-time media such as email and bulletin boards, not real-time media such as instant messaging. In the case of non-real-time media, they can refrain from their actions and also cancel their actions. Fourth, there should be more opportunities to improve interaction with parents at home, and a variety of programs should be offered to increase trust with friends.

## 5. Conclusions

The purpose of this study is to investigate the causes of cyberbullying among Korean adolescents. The purpose of this study, in particular, is to analyze the types, means, and forms of cyberbullying among adolescents. We were also trying to find the root cause of cyberbullying. For this study, the analysis was made based on the nationwide survey of cyberbullying status reports by the Korea Information Society Agency. In addition, two methods of analysis were performed, parametric and nonparametric, to induce reliable statistical analysis results. The conclusions of both methods of analysis were the same. The results of the analysis based on the data for the last three years are as follows. First, the statistically significant type of cyberbullying in adolescents is verbal violence. Second, a statistically significant means of cyberbullying is instant messaging. Third, the statistically significant form of cyberbullying occurs between individuals. On the other hand, the correlation between the interpersonal relationship of adolescents and the cyberbullying experience rates was analyzed, and the following results were obtained. First, the interaction between parents and children showed a statistically significant negative correlation with cyberbullying experience rates. That is, the higher the interaction with parents, the lower the cyberbullying experience rate. Second, the reliability of friend relationships showed a statistically significant negative correlation with cyberbullying experience rates. That is, the higher the reliability of friend relationships, the lower the cyberbullying experience rate.

The future research works of this study are as follows. First, it is necessary to analyze the causes of cyberbullying and its countermeasures in more depth. In other words, various interviews and in-depth analysis are needed. Second, it is necessary to develop teaching materials that can prevent and cure cyberbullying among adolescents. These teaching materials should be useful in real life by providing various examples as well as theories.

### Concluding Remarks

My main research area is information education, and specific research areas include information education for the disabled, gifted education in computer science, and information and communication ethics education. I am especially interested in etiquette in the cyberspace of adolescents in the field of information and communication ethics education, and I am also researching the causes and prevention of cyberbullying in cyberspace. The purpose of this research is to analyze the causes of cyberbullying in Korean adolescents based on national statistical data and present preventive measures.

## Figures and Tables

**Table 1:** Cyberbullying status of adolescents by type of cyberbullying.

Violence Type	2017	2018	2019
Overall	16.2	20.8	18.0
Verbal Violence	15.1	19.3	16.8
Defamation	2.9	4.6	3.6
Personal Information Leakage	2.2	2.2	1.9
Ostracism	1.5	1.9	1.8
Stalking	1.4	2.1	1.5
Coercion	0	1.5	1.0
Sexual Violence	1.0	1.5	1.0
Extortion	0.6	1.2	1.0

(Unit: %, multi responses allowed).

**Table 2:** Cyberbullying status of adolescents by type of cyber victimization.

Violence Type	2017	2018	2019
Overall	17.4	20.8	19.0
Verbal Violence	14.6	18.7	16.9
Defamation	5.4	6.1	6.2
Personal Information Leakage	2.6	2.5	2.1
Ostracism	1.5	2.4	1.7
Stalking	2.6	3.3	3.1
Coercion	0	1.6	1.1
Sexual Violence	1.9	2.7	2.2
Extortion	1.7	1.7	1.0

(Unit: %, multi responses allowed).

**Table 3:** Cyberbullying status of adolescents by means of cyberbullying.

Violence Means	2017	2018	2019
Instant Message	50.3	53.1	54.3
Online Game	41.5	38.6	36.9
SNS	34.1	23.9	25.9
Community	5.3	3.2	2.1
Personal Homepage	1.2	1.3	1.0
Email/Text	5.2	4.6	0.6

(Unit: %, multi responses allowed).

**Table 4:** Cyberbullying status of adolescents by means of cyber victimization.

Violence Means	2017	2018	2019
Instant Message	45.6	42.8	45.6
Online Game	38.8	42.3	38.9
SNS	35.3	28.3	35.3
Community	6.8	4.4	2.2
Personal Homepage	3.6	4.4	1.0
Email/Text	2.1	1.6	1.0

(Unit: %, multi responses allowed).

**Table 5:** Cyberbullying status of adolescents by means of cyberbullying.

Violence Forms	Multiple Assailants	One Assailant
2017	26.8	73.2
2018	22.5	74.5
2019	31.0	69.0

(Unit: %, multi responses allowed).

**Table 6:** Cyberbullying status of adolescents by means of cyber victimization.

Violence Forms	Multiple Victims	One Victim
2017	22.6	77.4
2018	25.2	74.8
2019	25.6	74.4

(Unit: %, multi responses allowed).

**Table 7:** Summary of statistical analysis for cyberbullying type.

Violence Type	Mean	SD	F	*p*
Verbal Violence	16.90	1.87	188.38 ***	0.000
Defamation	4.80	1.35		
Personal Information Leakage	2.25	0.26		
Ostracism	1.80	0.33		
Stalking	2.33	0.80		
Coercion	0.87	0.71		
Sexual Violence	1.72	0.68		
Extortion	1.20	0.43		

*** *p* < 0.001.

**Table 8:** Summary of statistical analysis for cyberbullying means.

Violence Means	Mean	SD	F	*p*
Instant Message	48.62	4.63	259.14 ***	0.000
Online Game	39.50	2.01		
SNS	30.47	5.07		
Community	4.00	1.85		
Personal Homepage	2.08	1.51		
Email/Text	2.52	1.93		

*** *p* < 0.001.

**Table 9:** Summary of statistical analysis for cyberbullying forms.

Violence Form	Mean	SD	F	*p*
Group-to-Person	25.62	3.14	−28.26 ***	0.000
Person-to-Person	73.88	2.76		

*** *p* < 0.001.

**Table 10:** Summary of statistical analysis for cyberbullying type (nonparametric case).

Violence Type	Mean	SD	Average Rank	Χ^2^(df)	*p*
Verbal Violence	16.90	1.87	45.50	36.48 *** (7)	0.0000
Defamation	4.80	1.35	39.17		
Personal Information Leakage	2.25	0.26	28.00		
Ostracism	1.80	0.33	20.17		
Stalking	2.33	0.80	26.00		
Coercion	0.87	0.71	8.17		
Sexual Violence	1.72	0.68	18.50		
Extortion	1.20	0.43	10.50		

*** *p* < 0.001.

**Table 11:** Summary of statistical analysis for cyberbullying means (nonparametric case).

Violence Means	Mean	SD	Average Rank	Χ^2^(df)	*p*
Instant Messaging	48.62	4.63	33.50	31.24 *** (5)	0.000
Online Gaming	39.50	2.01	27.50		
SNS	30.47	5.07	21.50		
Community	4.00	1.85	13.00		
Personal Homepage	2.08	1.51	7.08		
Email/Text	2.52	1.93	8.42		

*** *p* < 0.001.

**Table 12:** Summary of statistical analysis for cyberbullying forms (nonparametric case).

Violence Form	Mean	SD	Average Rank	Z	*p*
Group-to-Person	25.62	3.14	3.50	−2.88 ***	0.004
Person-to-Person	73.88	2.76	9.50		

*** *p* < 0.01.

**Table 13:** Parents’ involvement in internet use and their children’s cyberbullying experience rate.

Group	Cyberbullying Experience Rate	Victimization Experience Rate
Group of Parents with Low Involvement in Internet Use. (*n* = 3354)	18.8%	18.6%
Group of Parents with Higher Involvement in Internet Use (*n* = 268)	10.8%	14.9%

**Table 14:** Interaction between parents and children and children’s cyberbullying experience rate.

Group	Cyberbullying Experience Rate	Victimization Experience Rate
Group with Low Interaction between Parents and Children	26.9%	31.0%
Group with High Interaction between Parents and Children	17.0%	17.6%

**Table 15:** Reliability of friend relationships and cyberbullying experience rate.

Group	Cyberbullying Experience Rate	Victimization Experience Rate
Group of Friend Relationships with Less Reliability	26.5%	27.5%
Group of Friend Relationships with High Reliability	17.2%	18.2%

**Table 16:** Correlation of parents’ involvement in internet use and their children’s cyberbullying experience rates.

Subject	Cyberbullying/Victimization Experience Rate
Parents’ Involvement in Internet Use	−0.896 (0.104)

**Table 17:** Correlation of interaction between parents and children and the children’s cyberbullying experience rates.

Subject	Cyberbullying/Victimization Experience Rate
Interaction between Parents and Children	−0.970 * (0.030)

* *p* < 0.05.

**Table 18:** Correlation of reliability of the friend relationship and cyberbullying experience rate.

Subject	Cyberbullying/Victimization Experience Rate
Reliability of the Friend Relationship	−0.994 ** (0.006)

** *p* < 0.01.

**Table 19:** Reasons for cyberbullying.

Reason	2017	2018	2019
The other party did it first, to retaliate.	40.0	43.1	45.0
Angry with my opponent because I didn’t like it.	42.2	35.7	39.4
For fun, to relieve stress.	23.8	24.8	21.0
Contrary to my opinion, the other person said the wrong thing.	15.2	15.2	13.7
Just for no particular reason.	12.3	10.8	10.1
To hang out with friends, as they are around with friends.	6.2	7.3	7.2

(Unit: %, multi responses allowed).

**Table 20:** Post-abuse psychological state of cyberbullying perpetrators.

State	2017	2018	2019
Sorry and regretful	51.5	53.2	51.9
Worried about having problems	47.8	48.9	49.0
Considering that it is right	40.3	40.1	37.7
Interesting and fun	16.4	16.0	14.8
No feeling at all	32.1	29.7	32.1

(Unit: %, multi responses allowed).

**Table 21:** Cyberbullying victim’s response.

Response	2017	2018	2019
Blocking the other person or deleting or changing their own IDs or emails	35.0	37.8	36.6
The other person was asked to delete the abuse or to apologize directly	27.9	25.2	26.7
Report to the website concerned	20.0	20.8	21.8
Informing my friends, family, teacher, etc.	15.6	15.1	19.0
Informing the counseling and reporting center or reporting to the police	1.2	1.5	1.4
No action at all	30.7	30.5	28.2

(Unit: %, multi responses allowed).

**Table 22:** Reasons for not responding after cyber victimization.

Reasons	2017	2018	2019
I don’t think it’s a big deal	76.0	65.9	75.0
I thought it wouldn’t do much to report it	23.6	23.3	25.4
I thought I was at fault	27.1	23.0	19.9
I don’t know who the other person, who did harm to me, is	7.9	10.5	7.4
I’m afraid I’m going to get even more ostracized by my friends	4.8	7.1	3.9
I don’t know where to ask for help	4.4	5.1	3.9
For fear that the other person who hurt me might retaliate or threaten me	3.5	4.4	3.9
I’m sorry to have told you about the other person who hurt me	4.4	4.1	2.7

(Unit: %, multi responses allowed).

**Table 23:** Psychological states after victimization.

Reasons	2017	2018	2019
Desire for revenge to the other party	30.3	37.2	36.3
Depression, anxiety, and stress	21.1	22.8	20.9
Dislike for study/school	9.4	11.1	10.9
Difficulty in socializing	8.3	9.6	8.7
Suicidal desire	7.0	9.2	8.3
Not much thought	55.6	49.6	54.0

(Unit: %, multi responses allowed).

**Table 24:** Cyberbullying and cyber victimization experience rates by school and gender.

Rate	Year	School			Gender	
		Elementary	Middle	High	Boys	Girls
Both Rates	2019	24.2	32.4	24.3	27.8	25.9
	2018	24.4	32.9	31.0	32.5	25.8
	2017	18.4	31.0	24.6	29.3	18.9
Cyberbullying Experience Rate	2019	13.3	23.1	17.7	21.5	13.8
	2018	14.3	25.1	22.9	25.2	15.5
	2017	9.3	23.4	16.6	22.0	9.4
Cyber Victimization Experience Rate	2019	18.8	22.9	15.4	18.1	20.0
	2018	19.8	22.2	20.6	22.0	19.5
	2017	15.0	20.9	16.3	19.2	15.1

(Unit: %).
